# Absolute quantification of microparticles by flow cytometry in ascites of patients with decompensated cirrhosis: a cohort study

**DOI:** 10.1186/s12967-017-1288-3

**Published:** 2017-09-06

**Authors:** Cornelius Engelmann, Katrin Splith, Sandra Krohn, Adam Herber, Albrecht Boehlig, Stephan Boehm, Johann Pratschke, Thomas Berg, Moritz Schmelzle

**Affiliations:** 1 Section of Hepatology, Department of Internal Medicine, Neurology, Dermatology, University Hospital Leipzig, Liebigstraße 20, 04103 Leipzig, Germany; 2 Department of Surgery, Campus Virchow Klinikum, Charité-Universitätsmedizin Berlin, Augustenburger Platz 1, 13353 Berlin, Germany; 3 Klinische Virologie, Max von Pettenkofer-Institut, Medizinische Fakultät, Ludwig-Maximilians-Universität München, Pettenkoferstrasse 9a, 80336 Munich, Germany

**Keywords:** Cirrhosis, Ascites, Microparticles, MP

## Abstract

**Background:**

Microparticles (MPs) are small (<1 μm) cell membrane-derived vesicles that are formed in response to cellular activation or early stages of apoptosis. Increased plasma MP levels have been associated with liver disease severity. Here we investigated the clinical impact of ascites MPs in patients with decompensated liver cirrhosis.

**Methods:**

Ascites and blood samples of 163 patients with cirrhosis (ascites n = 163, blood n = 31) were collected between February 2011 and December 2012. MPs were obtained from ascites and from blood by two-step ultracentrifugation and quantified by flow cytometry. Quantitative absolute MP levels were correlated with clinical and laboratory baseline parameters as well as patient outcomes. Ascites microparticles were stained with antibodies against CD66b (neutrophils) and CD3 (lymphocytes) in a subgroup of 60 matched patients.

**Results:**

MPs were detected in all ascites and blood samples. Absolute ascites MP levels correlated with blood levels (r = 0.444, p = 0.011). Low ascites MP levels (<488.4 MP/μL) were associated with a poor 30-day survival probability (<488.4 MP/μL 71.1% vs. >488.4 MP/μL 94.7%, log rank p = 0.001) and such patients had a higher relative amount of ascites microparticles derived from neutrophils and lymphocytes. Low levels of ascites MPs, high MELD score and antibiotic treatment were independent risk factors for death within 30 days.

**Conclusions:**

Ascites MP levels predict short-term survival along with the liver function in patients with decompensated cirrhosis. Further studies which evaluate ascites MPs as disease specific biomarker with a validation cohort and which investigate its underlying mechanisms are needed. Neutrophils and lymphocytes contributed more frequently to the release of microparticles in patients with low ascites levels, possibly indicating an immune activation in this cohort.

**Electronic supplementary material:**

The online version of this article (doi:10.1186/s12967-017-1288-3) contains supplementary material, which is available to authorized users.

## Background

Microparticles (MPs) are small membrane-derived extracellular vesicles (<1 μm) that are shed by all cell types after cellular activation and during early stages of apoptosis. As they are formed by outward blebbing of the cell membrane, MPs contain cellular proteins, RNA and miRNA [1–3]. MPs are important messengers in intercellular communication and involved in various pathomechanistic processes. This explains why increasing plasma MP levels generally express disease activity and progression [4–9]. There are several diseases that are accompanied by vascular damage and therefore trigger the release of specific MP subtypes. In diabetes mellitus circulating platelet-derived MPs correlate with the degree of vascular damage, particularly if end-organ damage occurs [10]. MPs released by endothelial cells are involved in coronary artery disease [11]. The fact that well established therapies for coronary artery disease that improve patient outcomes, e.g. administration of statins, also reduce MP levels emphasizes the role of MPs as biomarkers of disease activity [12]. Importantly, MPs released in the context of inflammatory reactions or oxidative stress can be both the consequence as well as the active modulator of pathological processes [13, 14].

In liver diseases, plasma MP subtypes correlate well with intrahepatic inflammation in chronic hepatitis C and steatohepatitis [15, 16] as well as severity of liver failure [17]. If chronic liver diseases are not adequately treated, they most often progress to cirrhosis with typical complications such as ascites. Because all cell types shed MPs, they can also be expected to occur in body fluids other than plasma. There are studies demonstrating MPs in synovial fluid [18], pleural fluid [19] and urine [20]. Another source of MP release is the peritoneum. Mrvar-Brecko et al. [21] used electron microscopy to visualize the presence of MPs in a patient with peritonitis and ascites. The largest cohort was investigated by Press et al. [22]. Using flow cytometry, they have nicely shown that tumor-derived MPs are detectable in all ascites samples of 41 patients with ovarian carcinoma and eight patients with benign ovarian neoplasms. However, evidence for the presence of MPs in ascites of patients with cirrhosis is missing.

We therefore investigated whether MPs are detectable in ascites of patients with decompensated cirrhosis and correlated the quantification level with patients’ clinical presentation and outcome.

## Methods

### Study design

In line with the institutional standards of the Section of Hepatology, University Hospital Leipzig, Germany, every patient presenting with ascites between February 2011 and December 2012 was eligible for specimen banking and clinical data collection (n = 180). The study protocol conformed to the ethical guidelines of the 1975 Declaration of Helsinki and was approved by the local ethics committee. All patients gave written informed consent.

All patients with ascites due to decompensated liver cirrhosis were eligible for inclusion in the present analysis. Cirrhosis diagnosis was based on typical laboratory and morphological criteria. Exclusion criteria included immunosuppressive therapy, sepsis at baseline and malignant diseases other than hepatocellular carcinoma (HCC). Baseline was set at the first paracentesis of every patient after study inclusion. All further paracenteses were not considered for study analyses.

At baseline, the following parameters were assessed: etiology of cirrhosis, sex, age, body mass index (BMI), blood pressure, body temperature, drug history and clinical outcome, MELD (Model for End-Stage Liver Disease) score, liver and renal function tests [e.g. glomerular filtration rate (GFR)], international normalized ratio (INR), platelet count, white blood cell (WBC) count, serum sodium, transaminases [aspartate transaminase (AST), alanine transaminase (ALT)], gamma-glutamyl-transferase (GGT), C-reactive protein (CrP), serum albumin and in ascites leukocyte count, protein and albumin content. Spontaneous bacterial peritonitis (SBP) was diagnosed according to Wong et al. [23] if the ascites leukocyte count was elevated above 500/mm^3^. The ascites polymorphonuclear leukocyte (PMN) count, the gold standard for the diagnosis of SBP, was not available at this institution.

### Patient characteristics

The study included 163 patients with liver cirrhosis, of which 21 (12.9%) were diagnosed with hepatocellular carcinoma. The main cause of cirrhosis was alcoholic liver disease (71.8%). All further baseline characteristics are displayed in Table 1.

**Table 1:** Baseline parameters at index paracentesis

Variable	Value
Age (years)	59 (25–87)
Gender (male/female)	n = 121/n = 42 (74.2%/25.8%)
Etiology	
Alcoholic	n = 117 (71.8%)
NASH	n = 14 (8.6%)
Viral	n = 4 (2.5%)
Others	n = 28 (17.2%)
HCC	n = 21 (12.9%)
Child–Pugh class (A/B/C)^b^	n = 2/n = 60/n = 36 (2%/61.2%/36.7%)
MELD score^a^	16.9 (7–38)
INR	1.4 (1–2.9)
Total bilirubin level (μmol/L)	39.5 (2.9–609.1)
Creatinine level (μmol/L)	108 (38–509)
WBC count (exp9/L)	6.6 (1.2–49.7)
AST (μkat/L)	0.87 (0.1–8.9)
GGT (μkat/L)	1.8 (0.2–22.4)
CrP (mg/L)	25.4 (1.2–186.2)
Serum albumin (g/L)	30.4 (16–47.3)
Ascites protein content (g/L)	11.6 (2.7–50.9)
Ascites leukocyte count (/mm^3^)	180 (0–12,300)

Values are given in median (range) for continuous data and in absolute number (%) for discrete data

^a^MELD could not be calculated in 6/163 patients as at least one parameter was not available at paracentesis

^b^Child–Pugh score could not be calculated in 65/163 patients as at least one parameter was not available at paracentesis

Cirrhosis-associated complications were present at the time of index paracentesis with hepatic encephalopathy in 23 out of 163 patients (14.1%), hepatorenal syndrome in 41 out of 163 patients (25.2%) and hepatic hydrothorax in 17 out of 163 patients (10.4%). Twenty-one out of 163 (12.9%) had gastrointestinal bleeding within 4 weeks prior to paracentesis.

Forty-eight of 163 patients (29.4%) were on antibiotic treatment at the time of paracentesis. In total, 60 of 163 patients (36.8%) were treated with non-selective beta-blockers, 82 of 163 patients (50.3%) with proton-pump inhibitors and 82 of 163 patients (50.3%) with lactulose at the time of paracentesis.

SPB was diagnosed by means of high ascites leukocyte counts above 500/mm^3^ in 21 out of 163 patients (12.9%). Infections other than SBP occurred in 38 out of 163 patients (23.3%), 18 with urinary tract infections, eight with pneumonia, five with clostridium difficile-associated colitis, three with catheter-associated sepsis, one with soft tissue infection, one with pancreatic abscess and two with infection of unknown origin.

### Sampling of ascites and blood

Paracentesis was performed in all patients at baseline (index paracentesis) according to the EASL practice guidelines [24] if SBP was suspected or in patients with new onset or worsening of ascites. Ascites samples were collected under standard aseptic conditions. After skin disinfection, local anesthesia was injected. A 14-gauge cannula was inserted under ultrasound guidance. The first 50 mL of ascitic fluid were discarded to avoid contamination with skin microbiota. The subsequent 50 mL fraction was collected and 4 mL of ascitic fluid was immediately stored at −80 °C. Whole blood samples drawn on the day of paracentesis were available in a subgroup of 31 patients. Blood was centrifuged at 2500*g* and plasma was stored at −80 °C.

### Detection of ascites and plasma microparticles

Samples were isolated by two-step ultracentrifugation, as described previously [17]. Shortly, ascites fluid or plasma was thawed and 1 mL was ultracentrifuged at 10,000*g* for 30 min at 5 °C. In a second step the supernatant was ultracentrifuged by 100,000*g* for 90 min at 5 °C. After ascites fluid or plasma was discarded, plasma MPs were resuspended in 300 μL sterile filtered (0.2 µm) PBS, ascites MPs were resuspended in 200 µL sterile filtered (0.2 µm) PBS and all MPs were stored at −80 °C [17]. MPs were identified by flow cytometry. Analysis was performed on a FACS Canto II using DiVa software (BD Bioscience, San Jose, USA). MPs were identified by their characteristic forward and sideward scatter, which were set at logarithmic gain. MPs were simulated using different sizes of standard microbeads (0.5–1.0 μm, Invitrogen), and a microparticle gate (MP gate) was determined using these standards. The MP gate included 1.0 μm beads in its upper and outer corner so that it would contain all microparticles 1.0 μm or less. Events in the MP gate were further assessed with FACSFlow (BD Bioscience, San Jose, USA) and filtered (0.2 µm) PBS alone to distinguish true events from electronic noise. Event numbers of equal sample volumes were counted for 60 s with a flow rate of 120 µL/min. The measurements of all samples were performed on 1 day to avoid day-to-day variability of the flow cytometer. Furthermore, each sample was measured at least twice in random order to further minimize measurement variations. The total number of microparticles in the samples was calculated by multiplying the measured number of events with the ratio of total volume to measured volume. Values were reported as counts per microliter.

### Microparticle labeling and detection

In a subgroup of 60 patients ascites MPs were stained with antibodies against surface antigens which allow to allocate their origin to either neutrophils (CD66b) or lymphocytes (CD3). For that purpose 20, non-survivors with low ascites MP levels (<488.4 MP/μL) were matched with two survivors, one with low and one with high ascites MP level, according to the MELD score and age. Respectively, 50 µL MPs were incubated with labeled antibodies FITC-CD3 (UCHT1, lymphocytes, Biolegend, San Diego, USA), APC-CD66b (G10F5; granulocytes, Biolegend, San Diego, USA) for 15 min at RT. 450 µL of cold sterile filtered (0.2 µm) FACS buffer (PBS, 1% BSA, 0.1% NaN_3_) and 25 µL counting beads (Biolegend, San Diego, USA) were added. Analysis was performed on a FACS LSRFortessa using DiVa software (BD Bioscience, San Jose, USA) with the same approach and gates as described before. Prior measurements unbound antibody in FACS buffer as well as isotype controls (APC mouse IgM, FITC mouse IgG1; Biolegend, San Diego, USA) were run to exclude background and non-specific binding from real events. The number of positive MP was calculated relative to the number of all gated MPs by FACS (Additional file 1: Figure S1).

### Statistical analysis

Continuous variables were displayed as mean ± standard deviation or median with range, as appropriate. Categorical variables have been depicted as frequency and/or percentage. Mann–Whitney U test was used to compare continuous data and Chi square test for discrete data. A two-sided *p* value lower than 0.05 was considered statistically significant. Spearman-Rho correlation coefficient was used to display potential associations between variables. Survival was evaluated considering all events that occurred from the time of inclusion until 30 days after inclusion. Follow-up data were collected retrospectively by screening data gathered during clinically indicated visits. Patients or their relatives were contacted if follow-up data were not available. Data were censored at transplantation or at last patient contact. For survival analysis, Kaplan–Meier analysis was performed and the log-rank test was used for group comparison. All laboratory and clinical data were considered for Cox regression analysis to identify potential risk factors for short-term death (within 30 days). Factors that were significantly associated in univariate analysis were used for multivariate analysis.

## Results

### Detection of microparticles

**Fig. 1:**
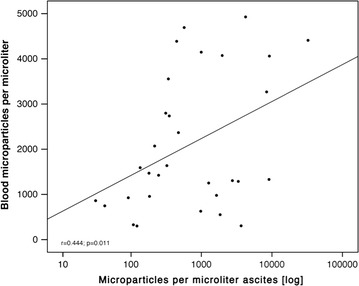
Correlation of microparticle levels between blood and ascites samples. Analysis was performed by Spearman-Rho. The ascites microparticles axis is scaled logarithmically

MPs were detected in all ascites (n = 163) and blood samples (n = 31) with a median MP count of 281.5 MP/μL in ascites (range 17.5–32,575.7) and 1469.7 (range 301.8–4926.3; (p = 0.357) in blood. Absolute MP levels in ascites samples correlated to individual MP levels in blood (r = 0.444, p = 0.011) (Fig. 1). A considerable number of patients showed higher MP levels in ascites than in blood samples (n = 11, 35.5%) and vice versa (n = 20, 64.5%), (Additional file 1: Figure S2).

**Fig. 2:**
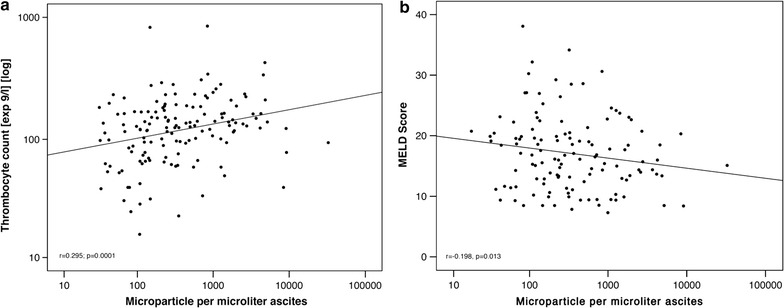
Correlation between ascites microparticle level and the thrombocyte count (**a**) as well as the MELD score (**b**) in blood. **a** The thrombocyte count in blood was weakly correlated with the level of microparticles per microliter ascites. Chart axis is scaled logarithmically. **b** The MELD score in blood was weakly correlated with the level of microparticles per microliter ascites. MELD axis is scaled logarithmically

Ascites MP levels correlated weakly with the thrombocyte count (r = 0.295, p = 0.0001) and inversely with the MELD score (r = −0.198, p = 0.013, Fig. 2a, b), but not with age, Child–Pugh score, serum albumin, white blood cell counts, AST, GGT, CRP, GFR, ascites protein content or the ascites leukocyte count. Blood MP levels also showed no significant correlation with the aforementioned parameters (Table 2). Ascites and blood MP levels at index paracentesis were not associated with the following baseline parameters: sex, presence of HCC, antibiotic treatment and non-selective beta-blocker use (Additional file 1: Table S1).

**Table 2:** Correlation between clinical baseline parameters and microparticles in ascites and blood

Variable	Ascites microparticles (n = 163)		Blood microparticles (n = 31)	
	Correlation coefficient (r)	Level of significance (p)	Correlation coefficient (r)	Level of significance (p)
Age (years)	0.042	0.590	0.144	0.440
Child–Pugh score	−0.092	0.390	−0.249	0.263
MELD score	−*0.198*	*0.013*	−0.318	0.990
Serum albumin (g/L)	0.056	0.584	0.286	0.196
Thrombocyte count (exp9/L)	*0.295*	*0.001*	0.140	0.461
WBC count (exp9/L)	0.047	0.570	0.067	0.726
AST (μkat/L)	0.071	0.423	0.011	0.953
GGT (μkat/L)	0.142	0.112	−0.009	0.956
CrP (mg/L)	−0.020	0.818	0.227	0.245
GFR (mL/min)	0.072	0.474	−0.040	0.862
Ascites protein content (g/L)	0.001	0.988	0.366	0.060
Ascites leukocyte count (/mm^3^)	0.050	0.951	0.121	0.516

Spearman-Rho correlation (r) was used for analysis

Values highlighted in italics are significant after multivariate analysis

### Association between microparticles and patient outcomes

**Fig. 3:**
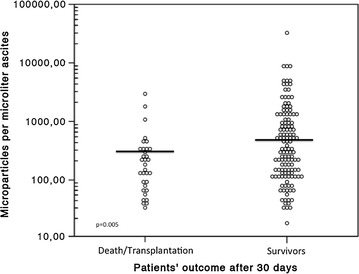
Total ascites microparticles levels in survivors and in patients who died or were transplanted. Although the range was wide, survivors showed significantly higher MP levels

Thirty-one of 163 patients (19%) died and two of 163 patients (1.2%) were transplanted within 30 days after study inclusion. Using the Cox regression analysis, ascites MP counts were shown to be significantly associated with the 30-day survival. There was an inverse correlation between risk of death and the ascites MP count (per 100 MP/µL: HR 0.928 (95% CI 0.862–0.999), p = 0.048). In patients who died or were transplanted within 30 days after paracentesis, ascites MP levels were significantly lower [median 180.5 (32.5–2851.6) MP/μL] as compared to patients surviving this period [325.1 (17.5–32,575.1) MP/μL, p = 0.005], (Fig. 3).

After ROC analysis, the best ascites MP quantification cut-off for predicting the 30-day mortality rate was 488.4 MP/μL with a sensitivity of 90.3% and a specificity of 44.7% and an area under the curve (AUROC) of 0.663 (95% CI 0.564–0.763) (p = 0.005).

**Fig. 4:**
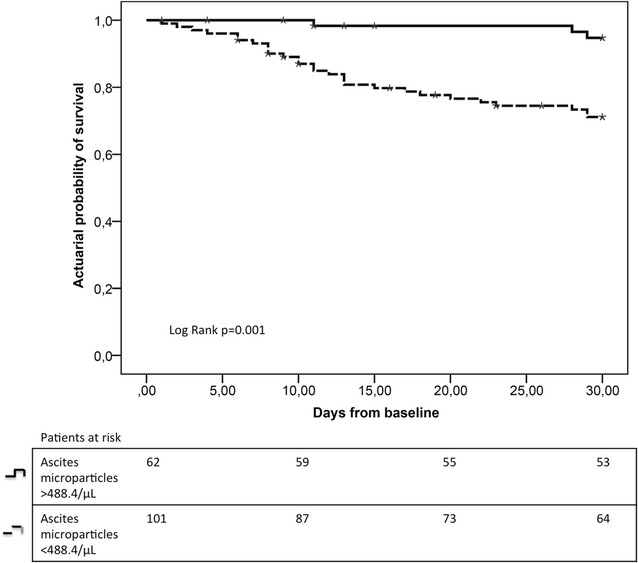
30-day survival analysis using Kaplan–Meier. Low levels of ascites microparticles were associated with poor short-term survival (30-day)

Kaplan–Meier analysis showed that patients with low levels of MPs in ascites (<488.4 MP/μL, n = 101) had a lower 30-day survival rate of 71.7% when compared to 94.7% in patients with high levels of MP in ascites (>488.4 MP/μL, n = 62), (p = 0.0001) (Fig. 4). In patients with high ascites MP levels the median bilirubin level [28.8 µmol/L (range 5.9–254.2) vs. 44.7 µmol/L (range 2.9–609.1, p = 0.04)] were slightly lower and the median thrombocyte count higher [141 exp9/L (range 34–864) vs. 116 exp9/L (range 16–826, p = 0.001)] than in patients with low ascites MP levels. All other baseline parameters were not different between both groups. Interestingly, blood MP levels were not associated with short-term survival.

Main cause of death was liver failure (Table 3) and sepsis. However, nine of 31 patients were lost to follow up after discharge from the hospital with regard to the cause of death.

**Table 3:** Cause of death (n = 31) taking into account the quantitative level of microparticles in ascites

Ascites MP level	Cause of death				
	Liver failure	Sepsis	Bleeding	Cardiac	Unknown
<488.4/μL (n = 101)	n = 10	n = 6	n = 2	n = 2	n = 8
>488.4/μL (n = 62)	n = 1	n = 1	n = 0	n = 0	n = 1

Liver failure was the main cause of death

### Factors associated with patients’ outcome

After univariate analysis, five factors were significantly associated with 30-day survival in this study cohort: thrombocyte count, MELD score, antibiotic treatment at paracentesis, ascites leukocyte count and low ascites MP counts (<488.4/μL). After adjustment with these risk factors by using a multivariate cox regression analysis three parameters remained being associated with death: low-level ascites MPs (<488.4/μL), the MELD score and antibiotic treatment at paracentesis (Table 4).

**Table 4:** Univariate and multivariate analysis using Cox regression analysis

Variable	Univariate analysis			Multivariate analysis		
	Hazard ratio	95% confidence interval	Level of significance (p)	Hazard ratio	95% confidence interval	Level of significance (p)
Thrombocyte count (exp9/L)	0.992	0.984–0.999	0.024	0.999	0.991–1.007	0.724
MELD score	1.167	1.097–1.241	<0.0001	*1.132*	*1.056–1.214*	*<0.0001*
Antibiotic treatment at paracentesis	3.422	1.518–7.718	0.003	*2.759*	*1.095–6.952*	*0.031*
Ascites leukocyte count (exp9/L)/100	1.019	1.001–1.037	0.039	0.997	0.958–1.038	0.900
Low-level ascites MPs (<488.4/μL)	6.552	1.991–21.561	0.002	*8.723*	*1.148–66.308*	*0.036*

High MELD score, antibiotic treatment at paracentesis and low-level ascites MP (<488.4 MP/μL) were independent risk factors for death after 30 days

Values highlighted in italics are significant after multivariate analysis

### Antibody labelling of ascites microparticles

**Fig. 5:**
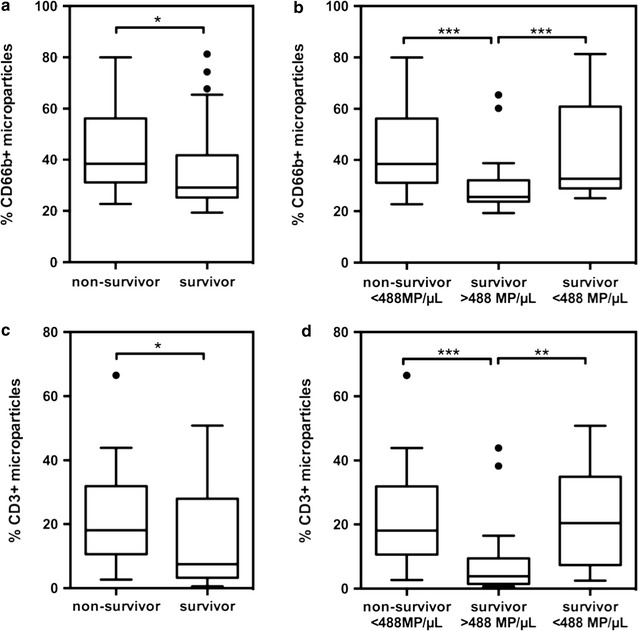
Relative amount of neutrophil (CD66b) and lymphocyte (CD3)-derived ascites microparticles compared between non-survivors and survivors (**a**, **c**) and according to the ascites MP levels (**b**, **d**). Non-survivors had a higher rate of CD66b positive (**a**) and CD3 positive (**c**) microparticles. **b** Patients with low ascites microparticle levels (<488.4/μL) had a higher relative amount of CD66b positive (**b**) and CD3 positive (**d**) ascites microparticles compared to patients with high ascites microparticle levels irrespective of their survival status. *p < 0.05; **p < 0.01; ***p < 0.001

Ascites MPs were labelled with antibodies capturing surface antigens from either neutrophils (CD66b) or lymphocytes (CD3) in a subpopulation of 60 matched patients. In general, the median relative amount of neutrophil-derived MPs was 32.2% (range 19.3–82.3) whereas the median relative amount of lymphocyte-derived MPs was 12.3% (range 0.6–66.5). There was a good correlation (r = 0.865; p < 0.0001) between both MP subsets in ascites and a weak correlation between the MELD score and neutrophil-derived MPs (CD66b r = 0.313, p = 0.016; CD3 0.158, p = 0.231). Conventional inflammatory parameters such as the CrP level and white blood cell count (CrP: CD66b r = 0.89, p = 0.526, CD3 r = 0.03, p = 0.83; WBC: CD66b r = −0.035, p = 0.79, CD3 r = −0.106, p = 0.424) and the leukocyte count in ascites (CD66b r = −0.052, p = 0.698; CD3 r = −0.106, p = 0.427) were not associated with the relative amount of immune cell-derived MP subsets. Patients who died with a low ascites MP count (<488.4/μL) had a higher relative amount of both, neutrophil and lymphocyte-derived MPs, than patients who survived or were transplanted [CD66b: median 43.1% (range 22.7–80) vs. median 29.1% (range 19.3–82.3), p = 0.01; CD3: median 18.1% (range 2.7–66.5) vs. 7.6% (range 0.6–50.8), p = 0.044)]. Moreover, the relative amount of immune cell-derived MPs of median 43.1% (range 25.1–81.3) for CD66b and median 22.4% (range 2.5–50.8) for CD3 in survivors with low ascites MP levels was comparable to non-survivors with low ascites MP levels (CD66b p = 0.578, CD3 p = 0.963) and significantly higher than in survivors with high ascites MP levels (CD66b median 25.6% (range 19.3–65.4), p = 0.006; CD3 median 3.9% (range 0.6–43.9), p = 0.0003) (Fig. 5a–d).

## Discussion

Plasma MPs, in particular cell specific subtypes, have been investigated as novel biomarkers for several pathomechanistic processes. We wanted to determine whether MPs are released into cirrhotic ascites in the first place and, if so, whether overall quantification levels correlate with clinical endpoints in cirrhosis.

This is the first report about ascites MPs in patients with liver cirrhosis. Our study indicates that MPs are ubiquitously present in cirrhotic ascites. This is in line with previous investigations that focused on MP diagnostics in ascites of patients with peritonitis and ovarian neoplasms. Those studies have shown that MPs can be found in peritoneal fluid irrespective of the diagnostic method used (electron microscopy or flow cytometry) and of the cause of ascites [20, 21].

Our survival analysis emphasized the clinical relevance of absolute ascites MP levels in cirrhosis. Patients with a cut-off level below 488.4 MP/μL died more frequently than patients with high levels (30-day survival: <488.4 MP/μL 71.1% vs. >488.4 MP/μL 94.7%, HR 8.571) along with the liver function and other clinical or laboratory parameters at baseline. The high rate of liver failure causing death indicated to some extent that patients with low ascites MP levels are in the final stages of cirrhosis. It is important to highlight that we observed an inverse correlation between ascites MP levels and patients’ prognosis whereas in the literature cell-specific plasma MP levels rise with disease progression. This might be explained by a formation of MP-associated immune complexes [25], which is a phenomenon observed in synovial fluid of patients with rheumatoid arthritis by using electron microscopy and a new generation of flow cytometers. Generally, those formations are highly active and pro-inflammatory and probably express an inflammatory systemic reaction [25]. These complexes would have been missed with the conventional flow cytometers that we used as particle size significantly increases (>1 µm) after complex formation. We gated only particles with a diameter smaller than 1 µm, so that we cannot exclude the presence of high volume complexes.

This theory is supported by the fact that the rate of MPs derived from neutrophils and lymphocytes are much higher in patients with low ascites MP levels and associated with the severity of liver dysfunction, displayed by the MELD score. This observation is well in line with the notion that end stage cirrhosis is accompanied by an inflammatory state [26, 27]. Nevertheless, further prospective studies are needed to substantially clarify the missing correlation with conventional inflammatory parameters as well as the underlying relationship between ascites MPs and patient outcomes. We suggest screening for MP complexes using new generation cytometers on the one hand and screening for further surface antigens in order to identify more cell types, which release MPs in ascites on the other hand. Subsequently performed ex vivo cell-specific stimulation tests to produce MPs can clarify if cell exhaustion is an actual problem in the peritoneal cavity.

Interestingly, the amount of MP production in ascites but also in blood from patients with cirrhosis seems to be highly variable, with MP levels in ascites ranging from 17.5 to 32,575.7 MP/µL, and in blood from 301.8 to 4926.3 MP/µL. This gives rise to at least two questions. The first is which host factors apart from inflammatory processes influence the release of MPs. The second is whether ascites MPs are derived from circulating blood cells or directly produced by autochthonous peritoneal cells. The latter might explain why we not only had a weak correlation between plasma and ascites MP levels but also a considerable number of patients with ascites MP levels higher than those in plasma. In this context it is important to emphasize that blood sample size was rather low potentially reducing its informative value. Nevertheless, the fact that there were no true correlations of overall MP levels, both in ascites and blood, with clinical parameters and immune cell MPs were upregulated in patients with high risk of death suggests that it is worthwhile again to focus on MP subsets in order to clarify the cause of overall ascites MP loss in high risk patients and the high variability. Notably, it is known that specific types of circulating MPs are involved in pathogenic processes such as endothelial dysfunction [4, 11, 23, 28, 29], coagulatory processes [9, 30] and inflammatory reactions [7, 31–33]. In recent years, there has been growing evidence that plasma MPs reflect disease severity in chronic liver diseases. Korneck et al. [16] investigated 67 patients with fatty liver disease and 42 patients with chronic hepatitis C and compared them to healthy individuals in terms of immune cell-derived plasma MP levels. We evaluated stem cell-derived plasma MP levels in acute as well as acute-on-chronic liver failure in rodents and humans (n = 10) [17]. Both studies showed that relative levels of immune cell- and stem cell-derived MPs were increased with heightened disease activity measured by transaminases and liver function tests in individuals with liver insufficiency. To what extend those results and others [34–38] related to circulating MPs can be extrapolated to ascites MPs is speculative at this stage, so that re-examinations are mandatory.

Our study has certain limitations. Due to the retrospective evaluation of all clinical data, some values were missing. Specific information concerning cause of death was not available for 9 of 31 patients who died during the observational period. Although the PMN count in ascitic fluid is the diagnostic gold standard for SBP, we used the ascites leukocyte count, because the ascites cell count could not be differentiated during the sample collection period. This might be a source of inaccuracy in terms of the relevance of infectious complications. However, the total leukocyte count has also been evaluated as a reliable diagnostic mean for SBP [22]. Moreover, ascites samples have not been centrifuged before freezing. Variation in temperature make cells (erythrocytes and leukocytes) explode and release membranes resembling MPs. However, cells and fragments of cells were removed by the first centrifugation step. If remaining cells had an influence on MP levels in our setting, we should have found a high correlation of the ascites leucocyte count with the MP levels, which is not the case. It is unclear as to whether MP levels correlated with the amount of ascites as a high ascites volume might be associated with low MP concentrations and vice versa. However, this parameters has not been documented. The fact that we were able to detect surface antigens from the original cell emphasizes that true MPs and not debris was captured by flow cytometry in ascitic fluid.

## Conclusions

In summary, absolute ascites MP levels represent a novel independent prognostic factor in patients with decompensated liver cirrhosis. Note that the detection of absolute MP levels from ascites samples was shown to be easy, reliable and cost-effective, which is important in the context of potential clinical implementation. Before MPs can be evaluated as biomarkers in cirrhosis, it is essential to clarify the mechanisms behind the association between overall ascites MP levels and clinical endpoints observed in this study. High levels of neutrophils and lymphocyte surface antigens in patients with low level ascites MPs suggests that immune reactions are involved in the pathomechanisms explaining the high risk of death in those patients.

## Additional file

**Additional file 1: Table S1.** Comparison of MP levels in ascites and blood according to baseline parameters at index paracentesis. Values are displayed in median (range) separated according to the presence or absence of parameters. **Figure S1.** Flow cytometry in ascitic fluid: Gating strategy and examples for different antigen expression profiles on ascites microparticles. **Figure S2.** Individual values for microparticles in plasma and ascites.

## Abbreviations

MP: microparticles
HCC: hepatocellular carcinoma
BMI: body mass index
MELD: model of end-stage liver disease
GFR: glomerular filtration rate
INR: international normalized ratio
WBC: white blood cell count
AST: aspartate transaminase
ALT: alanine transaminase
GGT: gamma-glutamyl-transferase (GGT)
CrP: C-reactive protein
SBP: spontaneous bacterial peritonitis
PMN: polymorphonuclear leukocyte
