# Supplementary material for: Absolute quantification of microparticles by flow cytometry in ascites of patients with decompensated cirrhosis: a cohort study
Source: J Transl Med. 2017 Sep 6;15:188. doi: 10.1186/s12967-017-1288-3 (PMC5586054; doi:10.1186/s12967-017-1288-3)
Supplement: Supplementary file 1 — Additional file 1: Table S1. Comparison of MP levels in ascites and blood according to baseline parameters at index paracentesis. Values are displayed in median (range) separated according to the presence or absence of parameters. Figure S1. Flow cytometry in ascitic fluid: Gating strategy and examples for different antigen expression profiles on ascites microparticles. Figure S2. Individual values for microparticles in plasma and ascites. [file 12967_2017_1288_MOESM1_ESM.docx]

Table S1: Comparison of MP levels in ascites and blood according to baseline parameters at index paracentesis. Values are displayed in median (range) separated according to the presence or absence of parameters.

| Variable | Median ascites microparticle level /μL (range) | Level of significance (p) | Median blood microparticle level /μL (range) | Level of significance (p) |
| --- | --- | --- | --- | --- |
| Sex  (female vs. male) | 218.6 (39.9 – 4844.4) vs. 321 (17.5 – 32575.1) | 0.317 | 1142 (748 – 4146.6) vs. 2070.6 (301.8 – 4926.3) | 0.295 |
| HCC  (no HCC vs. with HCC) | 269.1 (17.5 – 32575.1) vs. 324.6 (37.3 – 2851.6) | 0.964 | 1331.5 (301.8 – 4926.3) vs. 1853.75 (1591.5 – 2736) | 0.550 |
| Antibiotic treatment at paracentesis (no vs. yes) | 317.5 (30.9 – 32575.1) vs. 233.6 (32.5 – 8415.5) | 0.125 | 1530.6 (301.8 – 4926.3) vs. 1356.3 (748 – 3267.9) | 0.937 |
| Beta-blocker  (no vs. yes) | 240.4 (30.9 – 8415.5) vs. 400.3 (32.5 – 32575.1) | 0.080 | 1270.6 (301.8 – 4926.3) vs. 1614.2 (551.9 – 4690.8) | 0.208 |
| Proton-pump inhibitor (no vs. yes) | 281.4 (30.9 – 32.575.1) vs. 291 (31.7 – 5159.2) | 0.765 | 2766.8 (301.8 – 4926.3) vs. 1134 (306.1 – 4960.8) | 0.052 |


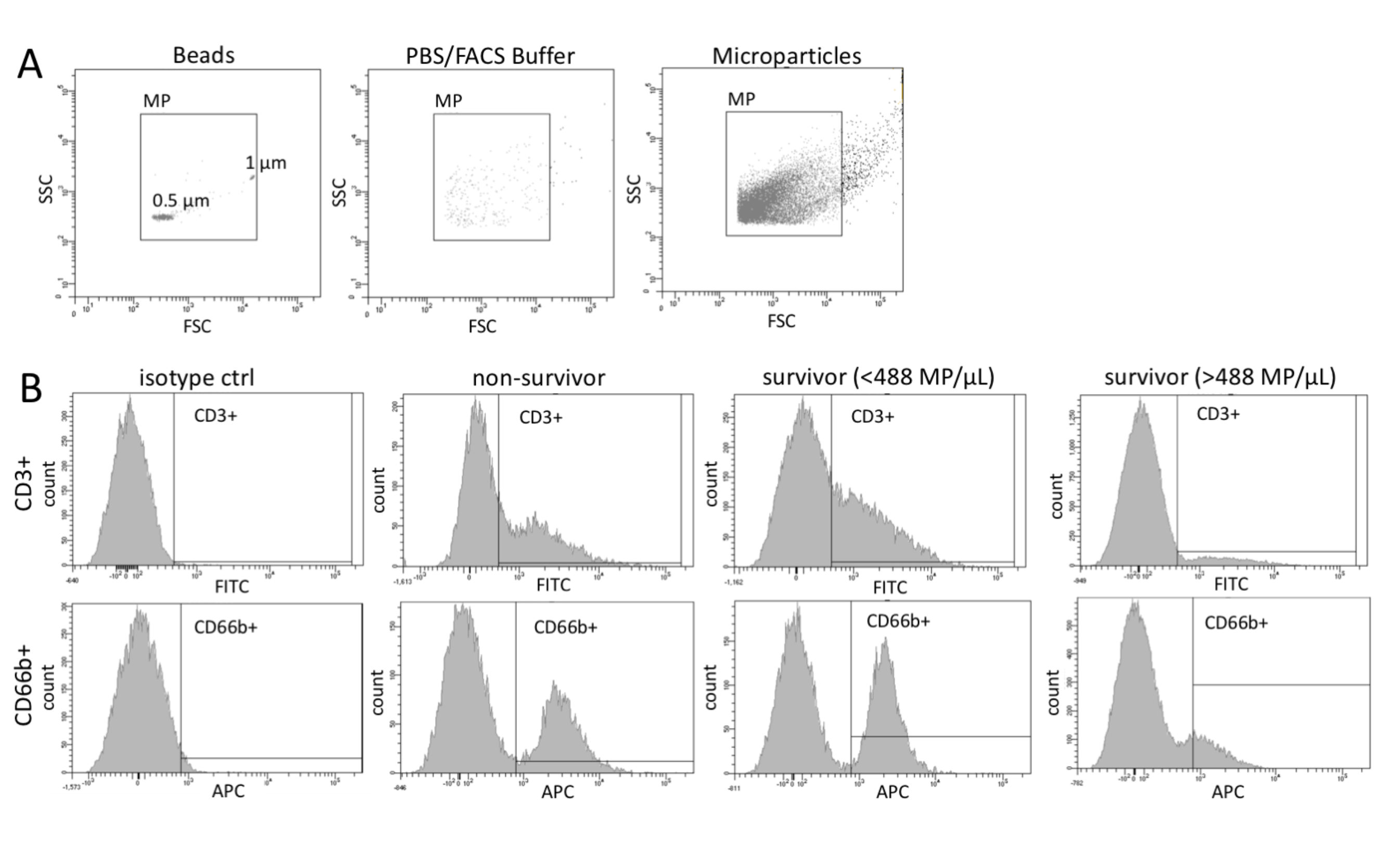


**Figure S1: Flow cytometry in ascitic fluid: Gating strategy and examples for different antigen expression profiles on ascites microparticles.**

Figure S2: Individual values for microparticles in serum and ascites
